# Comparative Study of Metabolic Tumor Volume in Multiple Myeloma and Related Plasma Cell Dyscrasias: 11C-Acetate PET vs. 18F-FDG PET

**DOI:** 10.7759/cureus.71605

**Published:** 2024-10-16

**Authors:** Momo Wakui, Junichi Tsuchiya, Chikara Kase, Kota Yokoyama, Masahide Yamamoto, Ukihide Tateishi

**Affiliations:** 1 Diagnostic Radiology and Nuclear Medicine, Institute of Science Tokyo, Tokyo, JPN; 2 Hematology, Institute of Science Tokyo, Tokyo, JPN

**Keywords:** 11c-acetate, 18f-fluorodeoxyglucose, metabolic tumor volume, multiple myeloma, positron emission tomography

## Abstract

Introduction

Accurate diagnosis of multiple myeloma (MM) and related disorders depends on imaging studies for lesion detection, which is crucial for treatment planning. The 18F-fluorodeoxyglucose (FDG) PET imaging system is well-established, with high sensitivity and specificity in identifying myeloma lesions. Additionally, 11C-acetate serves as an effective radiotracer for detecting MM lesions. Metabolic tumor volume (MTV) measured with 18F-FDG PET has been suggested as a prognostic factor in MM patients. This study aimed to compare the feasibility of measuring MTV in patients with myeloma-related diseases using 11C-acetate or 18F-FDG PET/CT.

Methods

We retrospectively reviewed six patients with MM - three with symptomatic MM and three with smoldering MM - and one patient with a myeloma-related disorder, all of whom underwent both 11C-acetate and 18F-FDG PET/CT scans. Using a dedicated workstation (PET-STAT; AdIn Research, Inc., Tokyo, Japan) equipped with a standardized uptake value (SUV)-based automated contouring program, we calculated the SUV for MTV. Bone areas with an SUV above thresholds of 2.0 or 2.5 were grouped accordingly.

Results

MTV detection in the whole body was significantly higher with 11C-acetate compared to 18F-FDG at both thresholds. For the 2.5 threshold, MTV was 129 ± 109 mL versus 3.93 ± 9.38 mL (p = 0.016), and for the 2.0 threshold, it was 316 ± 221 mL versus 12.1 ± 23.7 mL (p = 0.016).

Conclusions

MTV measurements were significantly higher in PET/CT using 11C-acetate compared to 18F-FDG. This finding highlights the potential superiority of 11C-acetate over 18F-FDG for assessing the MTV of lesions in patients with MM.

## Introduction

Multiple myeloma (MM) is the second most common hematologic malignancy, characterized by the infiltration of abnormal plasma cells in the bone marrow and the production of monoclonal immunoglobulins (M-proteins) [1,2]. Diagnosis of MM and related disorders is typically guided by the International Myeloma Working Group (IMWG) criteria, with MM always being preceded by monoclonal gammopathy of undetermined significance or smoldering MM [3,4]. According to the IMWG criteria, patients with MM exhibit one or more calcium elevation, renal failure, anemia, bone lesions (CRAB) symptoms or meet specific biomarker thresholds (≥60% clonal bone marrow plasma cells, serum free light chain ratio ≥100, or >1 focal lesion on MRI) [3]. Imaging plays a critical role in determining treatment strategies, as active MM is the primary treatment target.

Related plasma cell disorders, such as POEMS syndrome, share overlapping clinical features with MM but differ in disease characteristics. POEMS syndrome, a rare plasma cell disorder, is marked by polyneuropathy, organomegaly, endocrinopathy, monoclonal plasma cell disorder, and skin changes. While POEMS syndrome presents with CRAB-like symptoms, it typically has lower levels of bone marrow plasmacytosis than MM. Imaging studies are equally important in managing POEMS syndrome, especially for detecting plasmacytomas that may require radiation and systemic chemotherapy [5].

The conventional whole-body skeletal survey, considered the gold standard for screening bone lesions, has relatively poor sensitivity. Advanced imaging modalities, including CT, MRI, bone scintigraphy, and 18F-fluorodeoxyglucose (FDG) PET, have been introduced to improve lesion detection [6]. Among these, 18F-FDG PET is widely used for identifying osteolytic and other myelomatous lesions, with a sensitivity of approximately 80-90% and a specificity of 80-100% [7-10]. This technique is also valuable for differentiating active from precursor lesions and evaluating treatment response in MM patients [11-13]. However, about 30% of lesions detected by MRI are not visible on 18F-FDG PET/CT, and MRI is superior in identifying early-stage diffuse disease [14,15].

To address the limitations of 18F-FDG PET/CT, several other radiopharmaceutical tracers have been tested for MM diagnosis, including 18F-fluorodeoxy-L-thymidine, 18F-alpha-methyltyrosine, 11C-acetate, and others [1,16-18]. Of these, 11C-acetate is particularly promising due to its role in lipid and cholesterol synthesis, with increased uptake reflecting the heightened demand for membrane lipids in malignancies [19]. This tracer has been found to be upregulated in various cancers, including hepatocellular carcinoma, prostate cancer, and indolent lymphoma [20-22], and has demonstrated increased uptake in MM tumor cells [23].

Comparative studies have evaluated 11C-acetate PET/CT and 18F-FDG for detecting MM lesions. One study involving 35 patients found that 11C-acetate had higher sensitivity and specificity than 18F-FDG for symptomatic MM diagnosis [24]. Another study with 15 patients revealed a higher detection rate of diffuse plasma cell infiltrates using 11C-acetate. In 50 control subjects, the average maximum standardized uptake values (SUVmax) for the bilateral posterior iliac crests were 2.3 ± 0.7 for 11C-acetate and 2.0 ± 0.6 for 18F-FDG, suggesting a diagnostic advantage for 11C-acetate [25].

Metabolic tumor volume (MTV), a parameter for assessing tumor burden through PET imaging, is a useful prognostic tool for various malignancies. While MTV measured by 18F-FDG PET is proposed as a prognostic factor for MM, the feasibility of measuring MTV using 11C-acetate remains unexplored [26,27]. This study aimed to investigate the potential of 11C-acetate PET to measure MTV in patients with MM and POEMS syndrome compared to 18F-FDG PET.

## Materials and methods

Patients

The present study received approval from the Ethics Committee at Tokyo Medical and Dental University. All patients provided written informed consent prior to enrollment. Between October 2016 and February 2018, six patients who met the diagnostic criteria for MM [28] and one patient with POEMS syndrome were enrolled, all of whom underwent both 11C-acetate PET/CT and 18F-FDG PET/CT scans. Patients under 20 years of age, those who were pregnant or breastfeeding, and individuals with blood sugar levels exceeding 200 mg/dL at the time of the scan were excluded. Additionally, patients with malignancies unrelated to myeloma were also excluded from the study.

Data acquisition

The synthesis of 11C-acetate was achieved through the carbonation of the Grignard reagent. Specifically, 11C-carbon dioxide was reacted with 0.3 M methyl magnesium bromide, hydrolyzed using 1 M hydrochloric acid, and subsequently converted into 11C-acetic acid. This synthesis procedure adhered to the method established by Kruijer et al. [29,30].

Patients fasted for a minimum of six hours prior to the injection of 11C-acetate, and serum blood glucose levels were confirmed to be below 200 mg/dL. An intravenous administration of 3.7 MBq/kg of 11C-acetate was performed 20 minutes before the imaging scan. Imaging was conducted using an integrated PET/CT scanner (Celesteion®, Canon Medical Systems Corporation, Tokyo, Japan). CT data were acquired at 120 kVp and 40 mA, with a 550 mm field of view, a pitch factor of 16.0, a slice thickness of 2.0 mm, and a rotation time of 1.0 s. PET data were collected for 240 s per bed position with a 3 mm Gaussian filter and were reconstructed using time-of-flight, three-dimensional ordered subset expectation maximization (TOF 3D OSEM), covering the area from the top of the skull to the pelvis.

Image analysis

We utilized specialized software (PETSTAT; AdIn Research, Inc., Tokyo, Japan) to calculate SUV and MTV. Bone areas with SUVs exceeding thresholds of 2.0 or 2.5 were grouped, and the total volume within these tumor regions was automatically calculated. Calculations were performed separately using both thresholds of 2.0 and 2.5. Focal lesions were defined as areas of increased tracer uptake, with an SUVmax >2.0 or >2.5, correlating with CT abnormalities not attributable to other bone pathologies. Lesions were categorized into 12 anatomical regions: cervical, thoracic, and lumbar vertebrae; cranial; humerus; forearm bones; scapula; clavicle; ribs; pelvis; and femur. When a lesion extended into two or more bone regions, or when it could be automatically segmented from extra-skeletal uptake, the demarcation line was manually established by placing regions of interest. Measurements were conducted by two trained radiologists, who reached a consensus through discussion when necessary.

Statistical analysis

The Wilcoxon signed-rank test was employed to compare the MTVs measured using 11C-acetate and 18F-FDG PET. A p-value of less than 0.05 (two-tailed) was considered statistically significant. All statistical analyses were conducted using R software (version 3.5.1) and EZR (version 3.5.2) [31].

## Results

**Figure 1:**
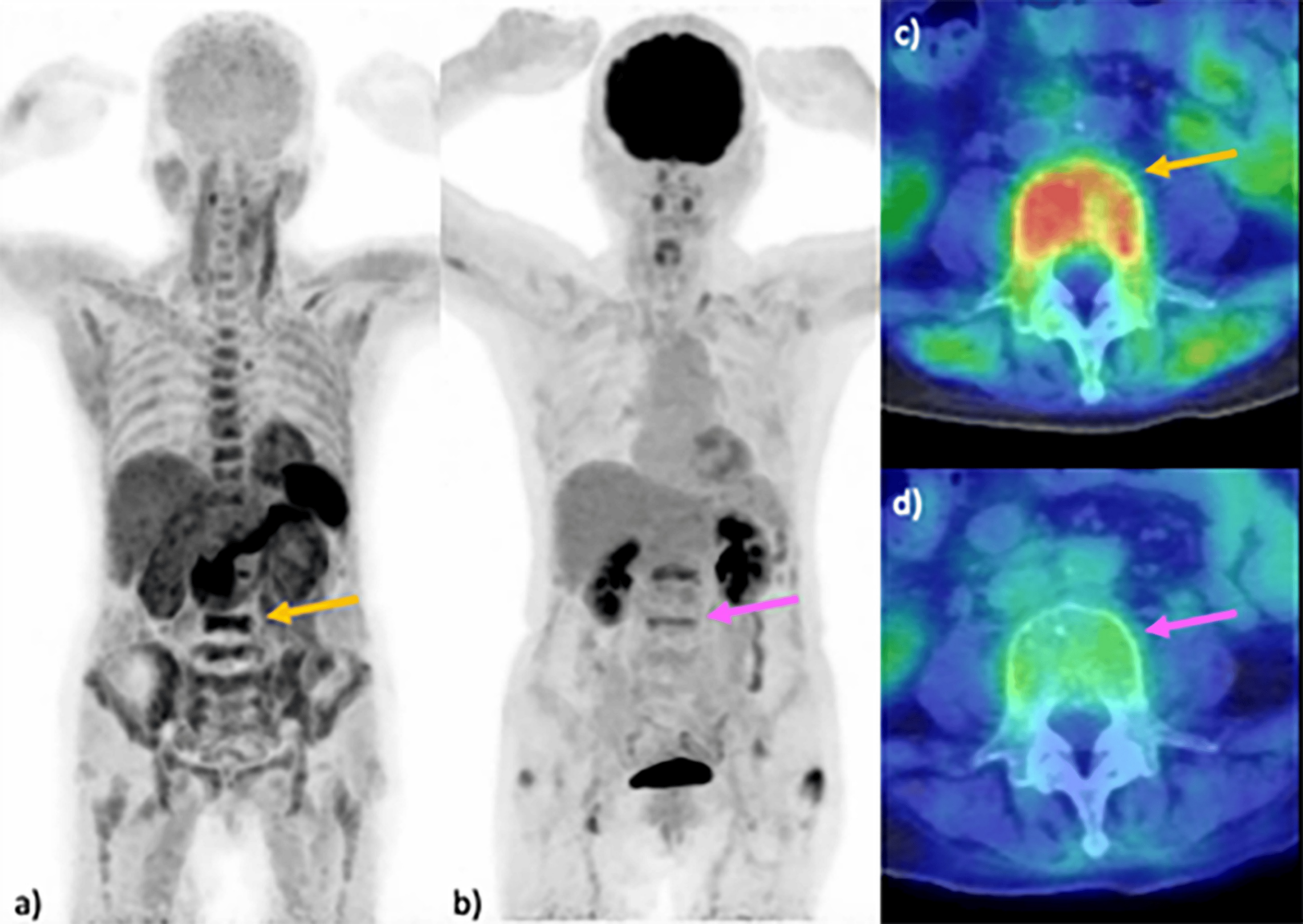
Comparison of PET imaging in MM: 11C-acetate versus 18F-FDG Imaging results from a 68-year-old woman with MM are presented. MIP images reveal multiple lesions in the vertebrae, including the L5 vertebral body (arrow) and pelvic bones, identified using 11C-acetate PET (a). Lesions in the lumbar vertebrae, including the L5 vertebral body (arrow), are detected by 18F-FDG PET (b). An axial fused PET/CT image utilizing 11C-acetate at the L5 vertebra demonstrates increased uptake in the vertebral body (SUVmax 4.0) (arrow) (c), in contrast to the relatively weak uptake observed in the 18F-FDG PET/CT image (SUVmax 2.0) (arrow) (d). FDG, fluorodeoxyglucose; MIP, maximum intensity projection; MM, multiple myeloma; SUV, standardized uptake value

**Figure 2:**
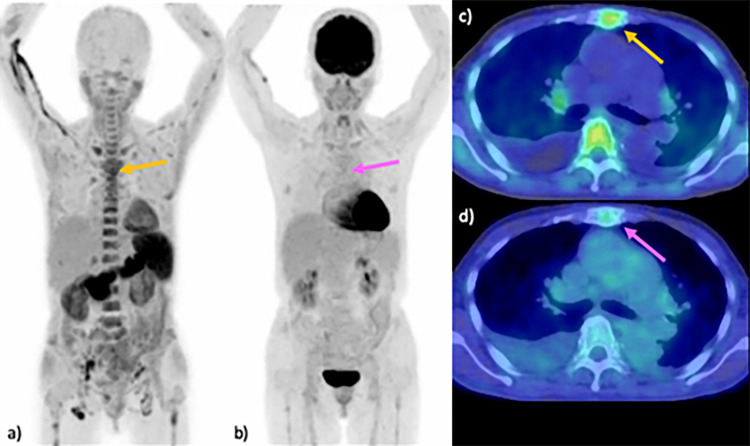
Comparison of PET imaging in POEMS syndrome: 11C-acetate versus 18F-FDG Imaging results of a 69-year-old woman with POEMS syndrome are presented. MIP images from the 11C-acetate PET scan revealed multiple lesions in the vertebrae and sternum (arrow) (a). Lesions in the thoracic vertebrae and sternum are detected using 18F-FDG PET (arrow) (b). An axial fused PET/CT image at the level of the thoracic vertebra demonstrates increased uptake of 11C-acetate in the sternal lesion (SUVmax 6.4) (arrow) (c), in contrast to minimal uptake observed with 18F-FDG (SUVmax 2.0) (arrow) (d). FDG, fluorodeoxyglucose; MIP, maximum intensity projection; SUV, standardized uptake value

Seven consecutive patients were included in this study, consisting of two men and five women, aged between 40 and 69 years. The cohort included three patients with symptomatic MM - one classified as stage II and two as stage I, according to the revised International Staging System [28] - three patients with smoldering MM (Figure 1), and one patient with POEMS syndrome (Figure 2). A summary of the cases and their respective MTVs is presented in Table 1.

**Table 1:** List of cases and MTVs measured with 11C-acetate and 18F-FDG FDG, fluorodeoxyglucose; MM, multiple myeloma; MTV, metabolic tumor volume; R-ISS, revised international staging system; sMM, smoldering multiple myeloma; SUV, standardized uptake value

Patient	Age (years)	Diagnosis	R-ISS stage	Threshold of SUV = 2.5		Threshold of SUV = 2.0	
				11C-acetate (mL)	18F-FDG (mL)	11C-acetate (mL)	18F-FDG (mL)
1	68	MM	II	345.44	26.9	728.44	69.83
2	62	sMM	NA	174.77	0	468.24	5.45
3	40	sMM	NA	2.27	0	64.97	0
4	59	sMM	NA	142.46	0	311.16	0
5	69	MM	I	43.55	0	92.39	6.02
6	63	MM	I	28.55	0	145.05	0
7	69	POEMS	NA	166.59	0	400.25	2.03

The whole-body MTV measured using 11C-acetate was significantly greater than that measured with 18F-FDG, with values of 129 ± 109 mL compared to 3.93 ± 9.38 mL, respectively (p = 0.016) at the SUV threshold of 2.5 (Table 2).

**Table 2:** MTVs of skeletal lesions with the SUV threshold of 2.5 measured in PET using 11C-acetate and 18F-FDG Data are presented as mean ± SD and range unless otherwise specified. FDG, fluorodeoxyglucose; MTV, metabolic tumor volume; SUV, standardized uptake value

Lesion location	11C-acetate (mL)	18F-FDG (mL)	p-value
Cervical	0.56 ± 0.68(0-1.91)	0 ± 0	0.1
Thoracic	36.2 ± 27.4 (1.00-73.1)	0 ± 0	0.016
Lumbar	37.2 ± 30.4 (0-77.11)	3.84 ± 9.41 (0-26.9)	0.036
Cranial	0.12 ± 0.21 (0-0.57)	0 ± 0	0.371
Humerus	0.48 ± 1.14 (0-3.26)	0 ± 0	0.371
Forearm	0.04 ± 0.07 (0-0.20)	0 ± 0	0.371
Scapula	0.08 ± 0.19 (0-0.53)	0 ± 0	1
Clavicle	0.68 ± 1.21 (0-3.39)	0 ± 0	0.371
Ribs	0.56 ± 1.15 (0-3.36)	0 ± 0	0.098
Sternum	3.44 ± 6.29 (0-18.2)	0 ± 0	0.181
Pelvis	47.8 ± 66.9 (0-207)	0.09 ± 0.21 (0-0.60)	0.036
Femur	1.88 ± 2.14 (0-5.62)	0 ± 0	0.1
Total	129 ± 109 (2.27-345)	3.93 ± 9.38 (0-26.90)	0.016

Lesions were detected in all seven patients using 11C-acetate PET, while only two patients had detectable lesions with 18F-FDG PET. At an SUV threshold of 2.0, the whole-body MTV was also significantly greater with 11C-acetate compared to 18F-FDG, measuring 316 ± 221 mL versus 12.1 ± 23.7 mL, respectively (p = 0.016) (Table 3).

**Table 3:** MTVs of skeletal lesions with the SUV threshold of 2.0 measured in PET using 11C-acetate and 18F-FDG Data are presented as mean ± SD and range unless otherwise specified. FDG, fluorodeoxyglucose; MTV, metabolic tumor volume; SUV, standardized uptake value

Lesion location	11C-acetate (mL)	18F-FDG (mL)	p-value
Cervical	4.66 ± 4.62 (0.10-13.1)	0 ± 0	0.016
Thoracic	89.62 ± 51.61 (11.1-160)	0.42 ± 0.73 (0-2.16)	0.016
Lumbar	81.78 ± 48.41 (2.26-137)	10.6 ± 24.1 (0-69.4)	0.016
Cranial	0.74 ± 1.22 (0-3.40)	0 ± 0	0.181
Humerus	1.37 ± 3.07 (0-8.88)	0 ± 0	0.371
Forearm	0.12 ± 0.26 (0-0.76)	0 ± 0	0.371
Scapula	0.4 ± 0.68 (0-1.90)	0 ± 0	0.181
Clavicle	1.63 ± 2.62 (0-6.85)	0.14 ± 0.35 (0-1.00)	0.181
Ribs	3 ± 3.31 (0-9.35)	0 ± 0	0.1
Sternum	6.66 ± 10.1 (0-29.8)	0.07 ± 0.17 (0-0.50)	0.059
Pelvis	120 ± 135 (8.97-424)	0.82 ± 1.99 (0-5.69)	0.016
Femur	6.13 ± 7.82(0-23.9)	0.05 ± 0.12 (0-0.33)	0.1
Total	316 ± 221 (65.0-728)	12.1 ± 23.7	0.016

Lesions were detected in all seven patients using 11C-acetate PET, while lesions were identified in only five patients with 18F-FDG PET at the SUV threshold of 2.0.

## Discussion

In the present study, MTV was significantly greater with 11C-acetate PET compared to 18F-FDG PET at SUV thresholds of 2.0 and 2.5. These findings underscore the superiority of 11C-acetate PET for measuring MTV in patients with myeloma-related diseases. This conclusion is consistent with previous reports highlighting 11C-acetate PET’s advantages over 18F-FDG PET in detecting lesions in myeloma patients [24,25]. In a prior study, a mouse model demonstrated that myeloma cells utilize extracellular acetate for anabolic metabolism, including the synthesis of cell membrane lipids [23]. Furthermore, this study indicated that the accumulation of 11C-acetate in bone corresponds with the concentration of myeloma cells in those areas. Given that prior research has established MTV measured by 18F-FDG PET/CT as a predictor of MM prognosis, the results of this study suggest that MTV measured with 11C-acetate PET/CT could also serve as a potential prognostic factor.

The optimal SUV threshold that accurately reflects tumor volume remains undetermined for both 18F-FDG PET and 11C-acetate PET. Previous studies employed an SUV threshold of ≥40% of the SUVmax in focal lesions with an SUVmax >2.5, demonstrating a significant correlation between MTV and diffuse bone marrow infiltration and disease progression [26]. At an absolute SUV threshold of 2.5, some lesions went undetected that were visible when the threshold was lowered to 2.0. However, automatic lesion demarcation proved challenging at this lower threshold, as lesions in different body areas became inseparable, potentially affecting measurement precision. Several prior studies using 18F-FDG PET/CT adopted a threshold of 2.5 to define myeloma lesions [26,32]. One study found no significant difference in average SUVmax values between 11C-acetate and 18F-FDG in 50 control subjects with normal bone marrow, with values of 2.3 ± 0.7 (range: 1.5 to 3.8) and 2.0 ± 0.6 (range: 1.2-3.2), respectively [25]. Therefore, a threshold of 2.5 or higher is deemed acceptable for detecting myeloma lesions with 11C-acetate PET/CT, leading us to define the optimal SUV threshold as 2.5 for measurements using this modality.

In this study, MTVs measured with 18F-FDG PET/CT were notably smaller compared to those reported in previous studies [26,27], likely because we did not include patients with advanced MM. Previous research has demonstrated the usefulness of 18F-FDG PET/CT in assessing POEMS syndrome [32-35]. In our cases, 11C-acetate PET/CT exhibited greater sensitivity in detecting osseous lesions associated with POEMS syndrome compared to 18F-FDG PET/CT, suggesting the potential value of 11C-acetate in evaluating this condition.

The present study has several limitations. First, the small number of patients included may limit the generalizability of the results. Second, the absence of a definitive lesion-based reference standard, such as biopsies, restricts the reliability of lesion evaluations. Due to the nature of this study conducted within routine clinical practice, performing invasive procedures was not feasible, resulting in no definitive lesion-based reference standard being established. Although we conducted patient-based assessments combining 18F-FDG PET/CT, MRI, and blood tests to evaluate active lesions, the accuracy of these assessments remains a limitation. Future studies that incorporate biopsies, MRI, and 18F-FDG PET/CT for lesion comparison will be essential to establish a more robust reference standard.

Third, while comparing visual and quantitative assessments is crucial, the lack of a definitive reference standard complicates the determination of one method’s superiority over another. SUV and MTV serve as objective measures to quantify visual information. Historically, visual assessments have classified lesions based on uptake patterns, while quantitative measures like SUVmax often attract more attention. We believe that MTV, which integrates uptake intensity and volume, offers a more comprehensive representation of lesion burden and aligns closely with radiologists’ visual assessments. Thus, we employed quantitative measures, including SUV and MTV, in this study to provide an objective representation of visual evaluations.

Lastly, manual demarcation of bone lesions may impair reproducibility. We utilized software to automatically extract areas with SUV above the threshold for MTV measurements, expecting minimal error for most lesions [36]. While there remains a possibility of overestimation in MTV measurements due to physiological uptake, we contend that this is more likely to occur with 18F-FDG than with 11C-acetate. To mitigate demarcation errors, we established consensus-based divisions of lesions among two observers. Further studies involving larger patient populations are needed to clarify the utility of MTV measurements as a prognostic factor for MM.

## Conclusions

11C-acetate PET/CT shows promise for measuring MTV in lesions of patients with MM and POEMS syndrome. This finding underscores the potential superiority of 11C-acetate over 18F-FDG in assessing MTV in these patient populations. Given 11C-acetate’s higher sensitivity for detecting bone lesions, it not only serves as a diagnostic tool but also holds promise for monitoring disease progression and treatment response. More accurate measurement of metabolic tumor burden with 11C-acetate may offer new prognostic insights, especially for patients with early or less aggressive disease, where 18F-FDG may be less effective.

Future studies involving larger cohorts are crucial for further validating these findings and establishing 11C-acetate PET/CT as a reliable imaging modality in clinical practice for MM and related plasma cell dyscrasias. Additionally, comparisons with other emerging radiotracers could refine the role of 11C-acetate in the comprehensive management of MM and associated conditions.
